# Single-cell transcriptomic sequencing data reveal aberrant DNA methylation in SMAD3 promoter region in tumor-associated fibroblasts affecting molecular mechanism of radiosensitivity in non-small cell lung cancer

**DOI:** 10.1186/s12967-024-05057-2

**Published:** 2024-03-16

**Authors:** Fushi Han, Shuzhen Chen, Kangwei Zhang, Kunming Zhang, Meng Wang, Peijun Wang

**Affiliations:** 1 Department of Medical Imaging, Tongji Hospital, School of Medicine, Tongji University, No. 389, Xincun Road, Putuo District, Shanghai, 200065 People’s Republic of China; 2 Institute of Medical Imaging Artificial Intelligence, Tongji University School of Medicine, Shanghai, 200065 China; 3 Department of Nuclear Medicine, Tongji Hospital, Tongji University School of Medicine, Shanghai, 200065 People’s Republic of China; 4 Department of Internal Medicine, Tongji Hospital, Tongji University School of Medicine, Shanghai, 200065 People’s Republic of China; 5 Department of Radiotherapy, Tongji Hospital, Tongji University School of Medicine, Shanghai, 200065 People’s Republic of China

**Keywords:** Single-cell sequencing, Non-small cell lung cancer, Radiosensitivity, Radiotherapy resistance, Tumor-associated fibroblasts, SMAD3, ITGA6

## Abstract

**Objective:**

Non-small cell lung cancer (NSCLC) often exhibits resistance to radiotherapy, posing significant treatment challenges. This study investigates the role of SMAD3 in NSCLC, focusing on its potential in influencing radiosensitivity via the ITGA6/PI3K/Akt pathway.

**Methods:**

The study utilized gene expression data from the GEO database to identify differentially expressed genes related to radiotherapy resistance in NSCLC. Using the GSE37745 dataset, prognostic genes were identified through Cox regression and survival analysis. Functional roles of target genes were explored using Gene Set Enrichment Analysis (GSEA) and co-expression analyses. Gene promoter methylation levels were assessed using databases like UALCAN, DNMIVD, and UCSC Xena, while the TISCH database provided insights into the correlation between target genes and CAFs. Experiments included RT-qPCR, Western blot, and immunohistochemistry on NSCLC patient samples, in vitro studies on isolated CAFs cells, and in vivo nude mouse tumor models.

**Results:**

Fifteen key genes associated with radiotherapy resistance in NSCLC cells were identified. SMAD3 was recognized as an independent prognostic factor for NSCLC, linked to poor patient outcomes. High expression of SMAD3 was correlated with low DNA methylation in its promoter region and was enriched in CAFs. In vitro and in vivo experiments confirmed that SMAD3 promotes radiotherapy resistance by activating the ITGA6/PI3K/Akt signaling pathway.

**Conclusion:**

High expression of SMAD3 in NSCLC tissues, cells, and CAFs is closely associated with poor prognosis and increased radiotherapy resistance. SMAD3 is likely to enhance radiotherapy resistance in NSCLC cells by activating the ITGA6/PI3K/Akt signaling pathway.

**Supplementary Information:**

The online version contains supplementary material available at 10.1186/s12967-024-05057-2.

## Introduction

Lung cancer is the most prevalent type of cancer globally and results in the highest number of deaths compared to other cancer types [1]. The 5-year survival rate for lung cancer is merely 18% [2]. Among the two types of lung cancer, namely small cell lung cancer (SCLC) and non-small cell lung cancer (NSCLC), NSCLC has the highest incidence rate, and patients with this type of lung cancer have a poor prognosis with low 5-year survival rates [3, 4]. Current strategies for lung cancer treatment include surgery, radiation therapy, and chemotherapy [5]. Nevertheless, due to the challenges in early diagnosis and malignant metastasis, the mortality risk for lung cancer patients remains remarkably high [6–8]. It is worth noting that the resistance to treatment caused by uncontrolled proliferation, invasion, and migration of lung cancer cells is attributed to various molecular pathways and mechanisms [9]. Radiation resistance is a significant concern in the treatment of non-small cell lung cancer (NSCLC) and is a leading cause of cancer-related mortality [10]. Despite its therapeutic potential, radiotherapy frequently fails to adequately control tumor growth, a failure partly attributed to the emergence of radioresistance [11]. In NSCLC, radioresistance emerges from a multifaceted process encompassing gene expression alterations, DNA damage repair pathway modifications, and changes in various signaling pathways [12]. Additional factors, such as tumor hypoxia and the presence of cancer stem cells, are also implicated in radioresistance development [13]. Consequently, an enhanced understanding of the mechanisms underpinning radioresistance in NSCLC is imperative for devising effective therapeutic strategies aimed at improving patient outcomes in this context [14]. Potential strategies include the development of novel radiosensitizers, targeted therapies, and combination treatment modalities to mitigate radioresistance and thereby enhance the efficacy of radiation therapy in NSCLC patients [15].

Cancer-associated fibroblasts (CAFs), as integral cellular constituents of the tumor microenvironment, play a pivotal role in regulating tumor progression and modulating tumor radioresistance. They exert their influence through the secretion of growth factors, chemokines, cytokines, signaling molecules, and components of the extracellular matrix [16]. Therapeutic approaches targeting the inhibitory modulation of CAF activity or their signaling pathways promise to enhance radiation therapy outcomes in NSCLC, thereby improving clinical responses in patients [17]. SMAD3, functioning as an intracellular signaling molecule for transforming growth factor-β (TGF-β) and activator receptors [18], has been implicated in regulating NSCLC cell proliferation, migration, and invasion. Notably, the silencing of SMAD3 has been observed to inhibit these cellular processes [19]. Furthermore, SMAD3 has been implicated in promoting radiotherapy and cisplatin resistance in NSCLC, mediated by RMRP [20].

The GSE20549 dataset reveals a positive correlation between SMAD3 and integrin subunit alpha 6 (ITGA6), a transmembrane glycoprotein adhesion receptor protein widely expressed in various tumors, known to facilitate cancer cell migration and invasion [21, 22]. Inhibition of ITGA6 has been shown to curtail NSCLC cell proliferation, colony formation, migration, and invasion, while inducing cell cycle arrest and apoptosis [23]. ITGA6 has been identified as a gene associated with radiation resistance, as evidenced by extensive cellular RNA-seq data from esophageal squamous carcinoma tissues and KYSE-150 cell lines of patients treated with radiotherapy [24–26]. The knockdown of ITGA6 in gallbladder cancer cells results in the inhibition of the phosphatidylinositol 3-kinase/protein kinase B (PI3K/Akt) pathway activity [27]. This pathway, known for its aberrant activation in cancers, plays a crucial role in tumorigenesis and progression, particularly in NSCLC, thus representing a vital target in combating this disease [28, 29]. Moreover, the activation of the PI3K/Akt pathway has been linked to enhanced radioresistance in NSCLC [12].

Therefore, we hypothesize that SMAD3 secreted by cancer-associated fibroblasts (CAFs) may regulate the downstream PI3K/Akt signaling pathway through its interaction with ITGA6, thereby affecting cell proliferation and survival and consequently influencing the sensitivity of non-small cell lung cancer (NSCLC) to radiation therapy. In this study, we aim to investigate the mRNA and protein expression levels of key molecules in the SMAD3/ITGA6 signaling pathway and relevant post-translational modifications using single-cell RNA sequencing (scRNA-seq) data, as well as in vitro and in vivo experiments. Additionally, by manipulating gene overexpression or silencing, we will explore the molecular mechanisms and biological functions regulated by the SMAD3/ITGA6 signaling pathway. Lastly, we will utilize animal models to further evaluate the potential role of SMAD3/ITGA6 in chemosensitivity of NSCLC, providing new theoretical evidence for the optimization of therapeutic strategies for NSCLC.

## Materials and methods

### Retrieval of public data for NSCLC research

NSCLC-associated datasets GSE2514, GSE18842, GSE21933, GSE31552, GSE44077, GSE20549, and GSE37745 were obtained from the GEO database. Specifically, dataset GSE2514 comprised 19 control samples and 20 NSCLC samples, GSE18842 included 45 control samples alongside 46 NSCLC samples, GSE21933 encompassed 21 control and 21 NSCLC samples, GSE31552 contained 68 control and 63 NSCLC samples, GSE44077 consisted of 66 control and 55 NSCLC samples, and GSE20549 featured 21 radioresistant NSCLC cell lines in contrast to 21 radiosensitive NSCLC cell lines. Additionally, gene expression data and corresponding clinical information for 196 NSCLC patients were procured from GSE37745 for the purpose of prognostic analysis.

The Kaplan–Meier plotter database was employed for the prognosis analysis of genes in NSCLC. The Human Protein Atlas database was also utilized to validate the protein expression in normal lung tissues and NSCLC tissues.

DNA methylation profiles (Illumina Human Methylation 450 K) from NSCLC tumor tissues in TCGA were downloaded from the UCSC Xena database. Concurrently, the methylation levels of genes in NSCLC and normal tissues were determined using the UALCAN and DNMIVD databases.

Differentially expressed genes (DEGs) in NSCLC were selected using the “limma” package in R software. The volcano plot was generated to depict the DEGs, with the threshold set at |log FC|> 1, FDR < 0.05, and P value < 0.05 [30].

### Analytical procedures for gene expression and protein–protein interactions in NSCLC

Differentially expressed genes (DEGs) in NSCLC were identified through the R “limma” package, with a significance threshold set at a p-value of less than 0.05. Subsequently, a volcano plot representing these DEGs was constructed.

Protein–protein interaction (PPI) analysis of the proteins encoded by target genes was conducted utilizing the STRING database. The visualization of this PPI network was achieved with Cytoscape 3.8.2 software. Within the PPI network, nodes were organized using the Degree method, and the top 15 nodes were selected for advanced analysis.

### Cox regression analysis and construction of nomogram

The GSE37745 dataset underwent univariate Cox regression analysis to discern prognostic genes for NSCLC. Hazard ratios (HR) and 95% confidence intervals (CI) for each gene were computed using the R “forestplot” package, adhering to a significance threshold of a p-value less than 0.05. Following this, both univariate and multivariate Cox regression analyses were executed to ascertain independent prognostic factors for NSCLC. A nomogram, based on the outcomes of the multivariate Cox regression analysis, was developed using the R “rms” package to forecast the 1-, 2-, and 3-year overall survival rates. The precision of the nomogram was evaluated through a calibration chart.

### Enrichment analysis and correlation assessment of DEGs

The NSCLC samples from the GSE37745 dataset were divided into two groups based on the median expression value of SMAD3: the high SMAD3 expression group and the low SMAD3 expression group. Gene Set Enrichment Analysis (GSEA) (http://software.broadinstitute.org/gsea/) was utilized to reveal the pathway differences between the high and low SMAD3 expression groups. The “c2.cp.kegg.v7.4.symbols.gmt” gene set from MSigDB was used as a reference gene set, with NOM p-val < 0.05 considered significantly enriched.

DEGs between the high and low SMAD3 expression groups were identified using the “limma” package, with a threshold of |log FC|> 1, FDR < 0.05, and P value < 0.05. Spearman’s method was employed to perform correlation analysis on the DEGs with a correlation coefficient > 0.4 and P < 0.05, yielding the SMAD3-related genes.

### Patient enrollment and sample collection

In this investigation, 120 patients (comprising 54 males and 66 females, aged between 29 and 84 years, with an average age of 62 years) were enrolled. These individuals underwent NSCLC surgery at Tongji Hospital, Tongji University School of Medicine, from January 2018 to January 2020. The study received approval from the Clinical Ethics Committee of Tongji Hospital, Tongji University School of Medicine (Approval No. SBKT-2023-052) and was conducted in strict adherence to the Declaration of Helsinki. Informed consent was duly obtained from all participants.

Tissue samples, both from NSCLC and adjacent normal areas, were excised from the enrolled patients and immediately frozen in liquid nitrogen. A portion of each tissue sample was fixed in 10% neutral buffered formalin and preserved in a − 80 ℃ freezer. None of the patients had received chemotherapy or radiotherapy prior to their inclusion in the study. The histopathological characteristics of the tumors were classified in accordance with the criteria of the eighth edition of the American Joint Committee on Cancer staging system manual [31]. Comprehensive information regarding the enrolled patients is detailed in Additional file 4: Table S1. Follow-up data were systematically collected, with overall survival defined as the duration from the date of surgery to either the date of death or the follow-up endpoint.

### Isolation of CAFs and normal tissue-associated fibroblasts (Nafs)

Tumor tissues and adjacent normal tissues (situated at least 2 cm from the tumor’s outer edge) from four NSCLC patients were surgically resected and dissected into pieces measuring 1–3 mm^3^. These tissue fragments were then digested with 1 mg/mL collagenase I (#C0130, Sigma) at 37 °C for 2 h. Following digestion, the solution underwent centrifugation at 1000 rpm for 5 min, was washed twice with PBS, and filtered through a 100 μm filter. The isolated cells were subsequently cultured in 10 cm dishes.

### Preparation of conditioned medium (CM) from fibroblasts

CAFs or NAFs were cultured in 75 cm^2^ bottles. After 48 h, upon reaching approximately 80% confluence, the medium was harvested and centrifuged at 3000 rpm for 30 min at 4 °C. The supernatant, designated as conditioned medium (CM), was collected and stored at − 80 °C, while the normal medium constituted fresh RPMI-1640 medium supplemented with 10% FBS [32].

### RT-qPCR

Total RNA was extracted from both cell lines and tissue samples utilizing TRIzol reagent (15,596–018, Solarbio). Subsequent to this, cDNA synthesis was performed using the PrimeScript™ RT-PCR kit (TaKaRa) for the purpose of mRNA detection. RT-qPCR analyses were conducted employing the SYBR Premix Ex TaqTM kit (TaKaRa) on the LightCycler 480 system (Roche Diagnostics, Pleasanton, CA). GAPDH served as the internal reference, and fold changes in gene expression were quantified using the relative quantification method (2^−△△Ct^ method) [33]. Primers, designed and synthesized by Shanghai General Co., Ltd. (Shanghai, China), are listed in Additional file 4: Table S2.

### Immunohistochemistry

Paraffin-embedded NSCLC tissue and mouse tumor sections, each 4 μm thick, underwent antigen retrieval and were subsequently blocked with 5% BSA (37,525, Thermo Fisher Scientific Inc., Waltham, MA) for 20 min. The sections were then incubated overnight at 4 °C with primary antibodies against SMAD3 (1:500, ab40854, Abcam Inc., Cambridge, UK), ITGA6 (1:500, ab181551, Abcam), and Ki67 (1:100, ab15580, Abcam). On the following day, sections were re-incubated with a biotinylated secondary antibody goat anti-rabbit IgG (1:1000, ab6721, Abcam) for 20 min and developed with DAB, followed by hematoxylin counterstaining. Microscopic examination was carried out (Leica-DM2500, Leica, Wetzlar, Germany) across five randomly selected fields from each section, with tan staining indicative of positive staining. Quantitative analysis was performed utilizing Image Pro-Plus 7.1 software (Media Cybernetics, Silver Spring, MD).

### Western blot

Tumor tissues and cells were lysed using enhanced RIPA buffer (P0013B, Beyotime, Shanghai, China) containing a 1% protease inhibitor cocktail for total protein extraction, after which protein concentration was determined. Proteins were separated by SDS-PAGE and electrotransferred onto PVDF membranes. The membranes, laden with protein, were then blocked with 5% BSA and incubated overnight at 4 °C with rabbit primary antibodies against SMAD3 (ab208182, 1:1000, Abcam), ITGA6 (ab181551, 1:2000, Abcam), PI3K (ab154598, 1:2000, Abcam), phosphorylated AKT (#9271, 1:1000, Cell Signaling Technologies [CST], Beverly, MA), AKT (#9272, 1:1000, CST), and GAPDH (ab181602, 1:10,000, Abcam). The following day, membranes were probed with an HRP-labeled secondary antibody IgG (rabbit, #7074, 1:5000, CST) for one hour at room temperature. ECL reagent (CPSOC, Sigma) was utilized for visualization of results on X-ray film (Z380164, Sigma), and ImageJ software was applied for quantitative analysis, with GAPDH as the internal reference.

### Methylation-specific PCR (MSP)

Modified DNA underwent purification and was subsequently subjected to methylation-specific PCR (MSP). For this assay, both methylation-specific and non-methylation-specific primers were utilized, along with 100 ng of bisulfite-modified DNA. PCR was carried out employing the GeneAmp DNA amplification kit (4,322,288, Applied Biosystems, Foster City, CA) and AmpliTaq Gold polymerase (4,298,813, Applied Biosystems). The PCR products were subsequently analyzed via agarose gel electrophoresis and visualized through ethidium bromide staining. The sequences of the primers used for MSP are detailed in Additional file 4: Table S3.

### Culture of cell lines

The human NSCLC cell lines H460 (HTB-177, ATCC, Manassas, VA), NCI-H2228 (CL-0570, Wuhan Procell Life Science & Technology Co., Ltd., Wuhan, China), NCI-H3122 (CBP60133, COBIOER BIOSCIENCES CO., LTD., Nanjing, Jiangsu, China), PC9 (CBP73272, COBIOER), H1229 (CRL-5803, ATCC), and H520 (CL-0402, Procell), along with the human bronchial epithelial cell line 16HBE (CL0249, Procell), were cultured in RPMI-1640 medium (12,633,012, Gibco, Grand Island, NY) supplemented with 10% FBS (26,140,079, Gibco). The cell culture was maintained in a 5% CO_2_ incubator at 37 °C.

### Cell treatment and lentiviral transduction

When H460 cells reached 75% confluence in 6-well plates (1 × 105 cells/well) after 24 h, they were treated with 50% NAF-CM or CAF-CM. Additionally, these cells were transduced with lentivirus carrying oe-NC, oe-SMAD3, oe-ITGA6, oe-SMAD3 + sh-NC, oe-SMAD3 + sh-ITGA6, or co-treated with CAF-CM and either sh-NC or sh-ITGA6.

H1229 cells underwent transduction with lentivirus carrying sh-NC, sh-SMAD3, sh-ITGA6, sh-SMAD3 + oe-NC, or sh-SMAD3 + oe-ITGA6. Following 8 h of transduction, the cells were exposed to varying doses of radiation (0, 2, 4, and 6 Gy), and 24 h later, subsequent experiments were conducted. Each experimental condition was replicated thrice. The mentioned lentivirus was procured from Shanghai Genechem Co., Ltd. (Shanghai, China), with the sequences presented in Additional file 4: Table S4.

### CCK-8 assay

Post-transduction, H460 and H1229 cells were seeded into 96-well plates at a density of 5 × 10^4 cells/well. They were subsequently exposed to varying radiation doses (0, 2, 4, and 6 Gy) for 48 h. Cell viability was determined using the CCK-8 kit (K1018, Apexbio). To each well, 10 μL of CCK-8 solution was added, and the cells were incubated for 2 h at 37 °C. The optical density (OD) value at 450 nm was measured using a Multiskan FC microplate reader (51,119,080, Thermo Fisher Scientific) to ascertain cell viability.

### Colony formation assay

Cells were plated in 6-well plates at a density of 200 cells per well and cultured for 24 h. Following this, the cells underwent irradiation at 6 Gy and were further cultured for an additional 14 days. Subsequently, the cells were fixed with 4% paraformaldehyde, stained with 0.5% crystal violet, and photographed. The number of colonies was counted.

### Transwell assay

A Transwell chamber (8 μm pore size; Corning Incorporated, Corning, NY, USA) in 24-well plates was utilized for the cell invasion experiment. Initially, 600 μL of RPMI-1640 medium with 20% FBS was added to the Transwell chamber lined with Matrigel and equilibrated at 37 °C for 1 h. NSCLC cells, post-transfection for 48 h, were resuspended in RPMI-1640 medium containing 10% FBS, and 100 μL of 1 × 10^9^/L cells were placed in the upper chamber. They were cultured at 37 °C with 5% CO_2_ for 24 h. Cells were then fixed with 4% methanol, stained with 0.1% crystal violet, and imaged under an inverted microscope (Olympus IX73, Olympus Optical Co., Ltd, Tokyo, Japan) in five randomly selected fields.

### Immunofluorescence staining

Cover glasses underwent washing in PBS and were blocked for 1 h in 10% BSA at 25 °C. The sections were then incubated with primary rabbit antibodies against γ-H2ax (1:400, #9718, Cell Signaling Technology), α-SMA (1:200, #19,245, Cell Signaling Technology), and FAP (1:200, #66,562, Cell Signaling Technology) at 4 °C for 12–16 h. This was followed by incubation with Alexa-Fluor 647-containing donkey anti-rabbit IgG (Thermo Fisher Scientific) for 1 h at 25 °C. Thereafter, sections were stained with DAPI (10 μg/mL, Sigma) for 15 min at 25 °C and observed under either an Olympus BX51 fluorescence microscope (Olympus) or a laser scanning confocal microscope (FV500; Olympus).

### Flow cytometry

Cell cycle progression and apoptosis in cells post-6 Gy X-ray radiation exposure were evaluated using a CytoFLEX-S flow cytometer (Beckman Coulter). This analysis employed the Annexin V-APC/PI Cell Apoptosis Detection kit and Cell Cycle Detection kit (KeyGen Biotech). Cells were harvested, washed with cold PBS, and then incubated with 5 μL Annexin V-APC and 1 μL PI working solution (100 μg/mL) in darkness for 15 min at room temperature. The obtained data were processed using FlowJo software. Apoptotic cells were identified, with the second and third quadrants indicating late and early apoptosis, respectively.

### Xenografts in nude mice

Eighty 4-week-old female SPF BALB/c nude mice (Beijing Vital River Laboratory Animal Technology Co., Ltd., Beijing, China) were accommodated individually in an SPF laboratory, maintained at 22–25 °C and 60–65% humidity, and subjected to a 12-h light/dark cycle. The mice had unrestricted access to food and water and were acclimatized for one week prior to experimentation. The animal experiments were conducted following approval from the Animal Ethics Committee of Tongji Hospital, Tongji University School of Medicine (Approval No. 2023-DW-SB-018), in compliance with the US National Institutes of Health's Guide for the Care and Use of Laboratory Animals.

The mice were subcutaneously injected with H460 cell suspensions (5 × 10^5^ cells/100 µL) treated with oe-NC, oe-SMAD3, oe-ITGA6, oe-SMAD3 + sh-NC, oe-SMAD3 + sh-ITGA6, or H460 cells equally mixed with NAFs, CAFs, CAFs + sh-NC, and CAFs + sh-ITGA6. Each group subsequently received 10 Gy X-ray radiation.

Irradiation was conducted using a Varian Clinac 600c, set at 250 cGy/min and positioned 80 cm from the skin [34]. Mice were anesthetized with isoflurane, shielded with lead to expose only the tumors to radiation. The irradiated tumor area was 1 cm larger than the tumor margin covered by the lead. Once the tumor volume reached 70 mm^3, the mice underwent 10 Gy X-ray radiation weekly for three weeks. After 28 days, the mice were euthanized under anesthesia with 1% sodium pentobarbital, and the tumors were excised and weighed. Each experimental setup was replicated three times. Tumor volume was calculated using the formula: volume = (length × width^2^)/2.

### TUNEL assay

Apoptosis within tumor tissues was detected using the TUNEL Apoptosis Detection Kit (Millipore, Billerica, MA). The sections were incubated in a 3% hydrogen peroxide solution in PBS for 20 min at room temperature, followed by incubation with a biotin labeling solution for 60 min at 37 °C in darkness. Subsequently, the sections were incubated with 50 μL Streptavidin-HRP working solution for 30 min at room temperature. The development process involved 0.2–0.5 mL DAB solution for 5–30 min, and images were captured under an inverted microscope from ten randomly selected fields per section. The count of apoptosis-positive cells (displaying brownish-yellow nuclei) and total cells (with blue-stained nuclei) was conducted. The apoptosis rate was calculated as the percentage of brownish-yellow cells to blue cells.

### Statistical analysis

For data processing, R statistical software (v4.1.1) (R Foundation for Statistical Computing, Vienna, Austria) and SPSS 21.0 (IBM Corp. Armonk, NY) were utilized. The correlation between SMAD3 gene expression and methylation levels at CpG sites was assessed using Spearman correlation analysis. Differences in methylation levels between the high and low SMAD3 expression groups were evaluated using Student’s t-test. Measurement data were represented as mean ± standard deviation. Comparisons between two groups were made using independent sample t-tests, while comparisons among multiple groups were conducted via one-way ANOVA, followed by Tukey’s post hoc tests. Time-based data were analyzed using repeated measures ANOVA. A p-value of less than 0.05 was considered indicative of statistical significance.

## Results

### Fifteen genes related to the radioresistance of NSCLC cells are identified based on the GEO datasets

To identify the key genes in NSCLC, we first performed differential analysis of the five datasets from GEO database (GSE2514, GSE18842, GSE21933, GSE31552 and GSE44077). The obtained results showed 3736 DEGs in the GSE2514 dataset, including 1986 upregulated DEGs and 1750 downregulated DEGs, 13,375 DEGs comprising 7185 upregulated DEGs and 6190 downregulated DEGs in the GSE18842 dataset, 6917 DEGs comprising 2811 upregulated DEGs and 3238 downregulated DEGs in the GSE21933 dataset, 7455 DEGs comprising 4125 upregulated DEGs and 3330 downregulated DEGs in the GSE31552 dataset, and 13,687 DEGs including 7949 upregulated DEGs and 5738 downregulated DEGs in the GSE44077 dataset (Additional file 1: Fig. S1A). Following intersection analysis of the up-regulated or downregulated DEGs from the five datasets, 637 upregulated DEGs (Additional file 1: Fig. S1B) and 610 downregulated DEGs (Additional file 1: Fig. S1C) were obtained.

**Fig. 1:**
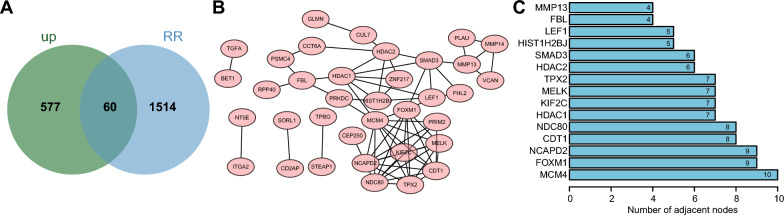
Screening of genes involved in the radioresistance of NSCLC cells**.** **A** Venn diagram of the upregulated DEGs in NSCLC tissues and radioresistant NSCLC cell lines. **B** PPI network diagram of the 60 genes related to radioresistance drawn by Cytoscape. **C** The top 15 genes related to radioresistance ranked by Degree value

Due to the significant impact of radioresistance on the survival of patients with non-small cell lung cancer (NSCLC), we conducted a differential analysis on the NSCLC cell lines in the GSE20549 dataset. We used a threshold of |log FC|> 1, FDR < 0.05, and P value < 0.05 to determine radioresistance and radiosensitivity. As a result, we identified 2795 differentially expressed genes (DEGs), including 1574 upregulated DEGs and 1221 downregulated DEGs (Additional file 1: Fig. S1D). Following Venn diagram analysis of the upregulated DEGs in NSCLC tissues and radioresistant NSCLC cell lines, 60 genes related to radioresistance were obtained (Fig. 1A). A PPI network was constructed using STRING database and visualized with Cytoscape software (Fig. 1B). The top 15 genes were displayed based on the Degree values: MCM4, FOXM1, NCAPD2, CDT1, NDC80, HDAC1, KIF2C, MELK, TPX2, HDAC2, SMAD3, HIST1H2BJ, LEF1, FBL and MMP13 (Fig. 1C). Collectively, 15 key genes related to radioresistance of NSCLC cells were identified from the GEO dataset.

### High SMAD3 expression is observed in CAFs, which is closely associated with poor prognosis in NSCLC patients

**Fig. 2:**
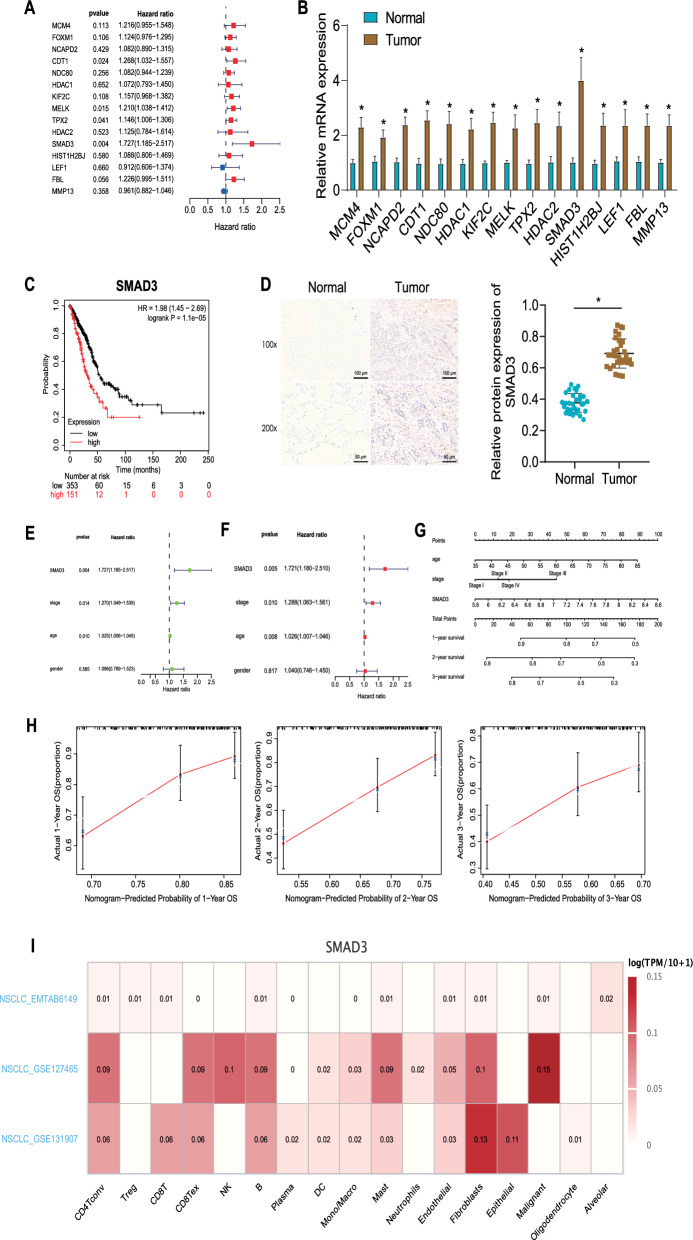
Identification of genes related to the prognosis of NSCLC patients. **A** Univariate Cox regression analysis of the GSE37745 dataset. The left shows the gene name, the middle is the *p* value, and the right indicates the risk rate distribution. Hazard ratio represents the risk rate, and a gene with the risk rate greater than 1 represents a high-risk gene, while less than 1, it represents a low-risk gene. **B** Expression of 15 genes in the clinical tissue samples and adjacent normal tissue samples of NSCLC patients (n = 120) measured by RT-qPCR. * *p* < 0.05. **C** Correlation between SMAD3 expression and overall survival in NSCLC patients analyzed by Kaplan–Meier plotter database. Red indicates SMAD3 high expression, black indicates low SMAD3 expression. **D** Immunohistochemistry of SMAD3 protein in normal lung tissues and NSCLC tissues (30 samples randomly selected from 120 samples). * *p* < 0.05. **E** Univariate Cox regression analysis confirmed SMAD3 as an independent prognostic factor of NSCLC. **F** Multivariate Cox regression analysis confirmed SMAD3 as an independent prognostic factor of NSCLC. **G** A nomogram composed of SMAD3 expression, age, and tumor stage. **H** Accuracy of the predicted 1-, 2- and 3-year survival rate determined by a calibration curve based on the nomogram. **I** SMAD3 enrichment analyzed by the NSCLC-related scRNA-seq dataset from the TISCH database. The abscissa represents the cell type, the ordinate represents the dataset and color scale indicates expression, with darker color corresponding to higher expression

To determine the correlation of the obtained 15 radioresistant genes with the prognosis of NSCLC patients, we performed univariate Cox regression analysis on the GSE37745 dataset, and found that SMAD3 had the most significant prognostic value in NSCLC, representing a high-risk gene (Fig. 2A). RT-qPCR data confirmed that the expression of the above 15 genes increased to varying degrees, and the expression of SMAD3 was significantly increased in clinical tissue samples compared with adjacent normal tissue samples (Fig. 2B). The analysis results of the Kaplan–Meier plotter database showed that the high expression of SMAD3 was inversely correlated with the overall survival of NSCLC patients (Fig. 2C). In addition, immunohistochemistry results illustrated that the protein expression of SMAD3 was upregulated in NSCLC tissues (Fig. 2D).

Univariate Cox regression analysis results showed that SMAD3 expression (*p* = 0.004, HR = 1.727, 95% CI 1.185–2.517), tumor stage (*p* = 0.014, HR = 1.270, 95% CI 1.049–1.539), and age (*p* = 0.010, HR = 1.025, 95% CI 1.006–1.045) considerably affected the overall survival of NSCLC patients (Fig. 2E). Besides, multivariate Cox regression analysis data indicated that SMAD3 expression (*p* = 0.005, HR = 1.721, 95% CI 1.180–2.510), tumor stage (*p* = 0.010, HR = 1.288, 95% CI 1.063–1.561), and age (*p* = 0.008, HR = 1.026, 95% CI 1.007–1.046) could serve as independent prognostic factors for NSCLC (Fig. 2F). A nomogram was constructed based on these three independent prognostic factors to predict the survival rate of NSCLC patients (Fig. 2G), which explained that the predicted 1-, 2- and 3-year survival rate was highly consistent with the actual survival rate (Fig. 2H). This indicates that high expression of SMAD3 is associated with the poor prognosis in NSCLC patients.

CAFs have been confirmed as a major component of the tumor microenvironment, which can induce tumor radioresistance [32]. Therefore, we sought to further verify the correlation between SMAD3 expression and CAFs. Analysis of the NSCLC-related scRNA-seq dataset from the TISCH database suggested that SMAD3 was highly enriched in CAFs (Fig. 2I).

Accordingly SMAD3 served as an independent prognostic factor, and its high expression is associated with poor prognosis in NSCLC patients. In addition, high SMAD3 expression was found in CAFs in the tumor microenvironment.

### High SMAD3 expression correlates with DNA hypomethylation at its promoter

**Fig. 3:**
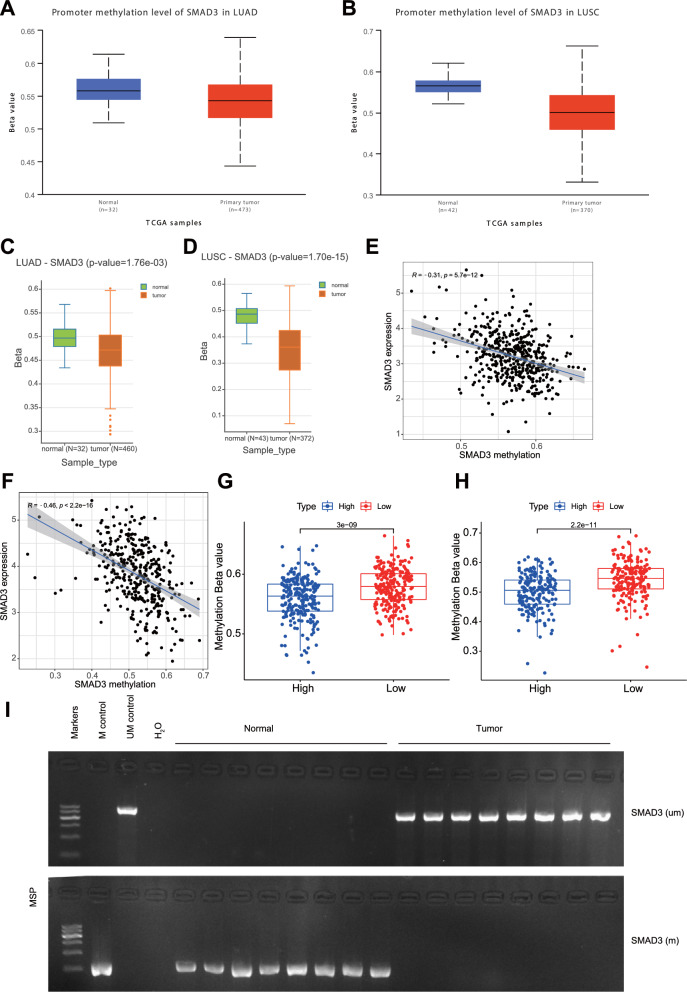
Increased expression of SMAD3 in NSCLC is correlated with its promoter hypomethylation. **A** The DNA methylation level of the SMAD3 promoter region between normal tissues and LUAD tissues analyzed by the UALCAN database. **B** The DNA methylation level of the SMAD3 promoter region between normal tissues and LUSC tissues analyzed by the UALCAN database. **C** The DNA methylation level of the SMAD3 promoter region between normal tissues and LUAD tissues analyzed by the DNMIVD database. **D** The DNA methylation level of the SMAD3 promoter region between normal tissues and LUSC tissues analyzed by the DNMIVD database. **E** Correlation between SMAD3 expression and DNA methylation of the SMAD3 promoter region in TCGA-LUAD (n = 465). **F** Correlation between SMAD3 expression and DNA methylation of the SMAD3 promoter region in TCGA-LUSC (n = 370). **G** The DNA methylation level of the SMAD3 promoter region between the SMAD3 high (n = 233) and low expression groups (n = 232) in TCGA-LUAD. **H** The DNA methylation level of the SMAD3 promoter region between the SMAD3 high (n = 185) and low expression (n = 185) groups in TCGA-LUSC. **I** The DNA methylation level of the SMAD3 promoter region in promoter region in the tumor tissues of NSCLC patients detected by MSP-PCR (8 samples randomly selected from 120 samples)

Next, we moved to determine whether dysregulation of SMAD3 expression is correlated with its altered methylation status. Analysis using the UALCAN (Fig. 3A, B) and DNMIVD databases (Fig. 3C, D) showed significantly lower DNA methylation levels of the SMAD3 promoter region in lung adenocarcinoma (LUAD) and lung squamous cell carcinoma (LUSC) tissues compared to normal tissues.

Furthermore, increased SMAD3 expression was found to be correlated with the SMAD3 promoter hypomethylation in both LUAD and LUSC tissues (Fig. 3E, F). Eighteen of the 29 promoter CpG sites in LUAD were negatively correlated with SMAD3 expression (Additional file 2: Fig. S2A, C), and 17 of the 29 promoter CpG sites in LUSC were adversely correlated with SMAD3 expression (Additional file 2: Fig. S2B, D). In addition, DNA methylation level of the SMAD3 promoter region was lower in the SMAD3 high expression group than that in the SMAD3 low expression group (Fig. 3G, H).

Meanwhile, MSP results showed that DNA methylation level of the promoter region of SMAD3 was decreased in tumor tissues compared with adjacent normal tissues (Fig. 3I).

Therefore, high expression of SMAD3 in NSCLC correlates with its promoter hypomethylation.

### SMAD3 promotes radioresistance of NSCLC cells by activating the ITGA6/PI3K/Akt pathway

**Fig. 4:**
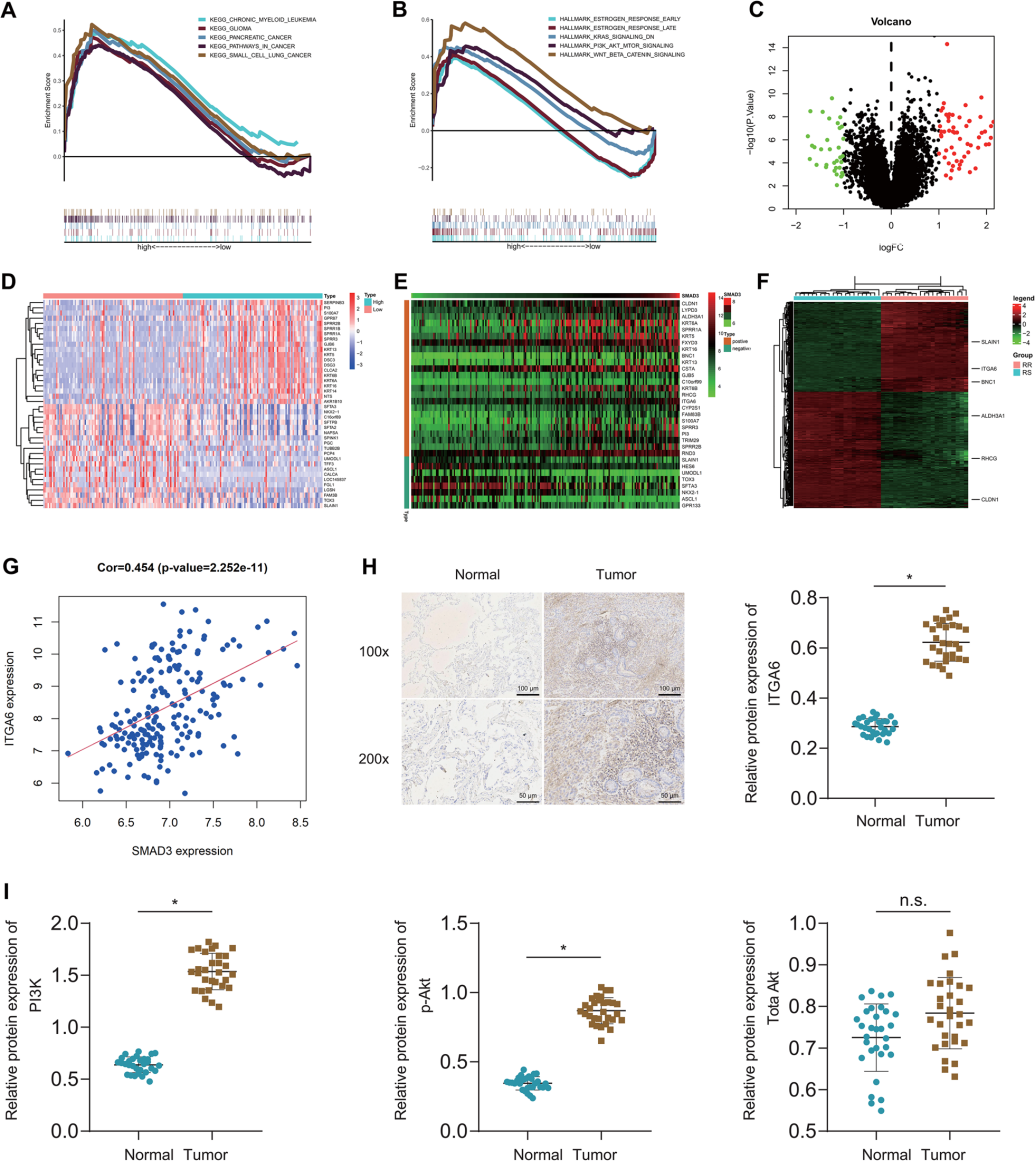
SMAD3 enhances the radioresistance of NSCLC cells via ITGA6/PI3K/Akt pathway activation. **A** Analysis of the function of SMAD3 using GSEA based on the KEGG gene set. Different curves indicate different pathway names. **B** Analysis of the function of SMAD3 using GSEA based on the Hallmark gene set. Different curves indicate different pathway names. **C** A volcano map of the DEGs between the SMAD3 high (n = 98) and low expression groups (n = 98). Green indicates the downregulated genes, red indicates the upregulated genes, and black indicates un-differentially expressed genes. **D** A heat map of the top 20 DEGs between the SMAD3 high and low expression groups. Color scale from red to blue indicates gene expression from high to low. **E** A heat map of SMAD3-related genes. Color scale from red to green indicates gene expression from high to low. **F** A heat map of the DEGs in the GSE20549 dataset, with 6 SMAD3-related genes labeled. Color scale from red to green indicates gene expression from high to low. **G** Correlation of SMAD3 expression with ITGA6 expression in the GSE37745 dataset (n = 196). **H** The protein expression of ITGA6 in tumor tissues of NSCLC patients determined by immunohistochemistry. **I** Western blot of PI3K/Akt pathway-related proteins in tumor tissues of NSCLC patients (30 samples randomly selected from 120 samples). * *p* < 0.05

Subsequently, this study aimed to elucidate the potential regulatory mechanism of SMAD3 in NSCLC. Analysis of the function of SMAD3 using GSEA showed that high SMAD3 expression was related to multiple types of cancer including NSCLC, and cancer-related pathways such as PI3K/Akt/mTOR and KRAS (Fig. 4A, B).

In addition, 95 DEGs were screened between high SMAD3 expression group and low SMAD3 expression group following differential analysis (Fig. 4C, D), of which, 32 DEGs were correlated with SMAD3 expression, as revealed by Spearman correlation analysis (Fig. 4E). In addition, 6 genes were found in the radiotherapy-related dataset GSE20549 (Fig. 4F). Among them, ITGA6 was highly expressed in radioresistant samples and positively correlated with SMAD3 expression (Fig. 4G). Additionally, ITGA6 has been demonstrated to enhance radioresistance through the PI3K/Akt signaling pathway [35]. Therefore, we hypothesized that SMAD3 may promote radioresistance of NSCLC cells by activating the ITGA6/PI3K/Akt pathway.

Immunohistochemistry results suggested increased ITGA6I expression in tumor tissues (Fig. 4H). Western blot results showed that PI3K protein expression and Akt phosphorylation level were increased while total Akt protein expression remained unchanged in tumor tissues compared with adjacent normal tissues (Fig. 4I).

These results suggest that SMAD3 may promote radioresistance of NSCLC cells by activating the ITGA6/PI3K/Akt pathway.

### DNA hypomethylation of the SMAD3 promoter region in CAFs increases the radioresistance of NSCLC cells

**Fig. 5:**
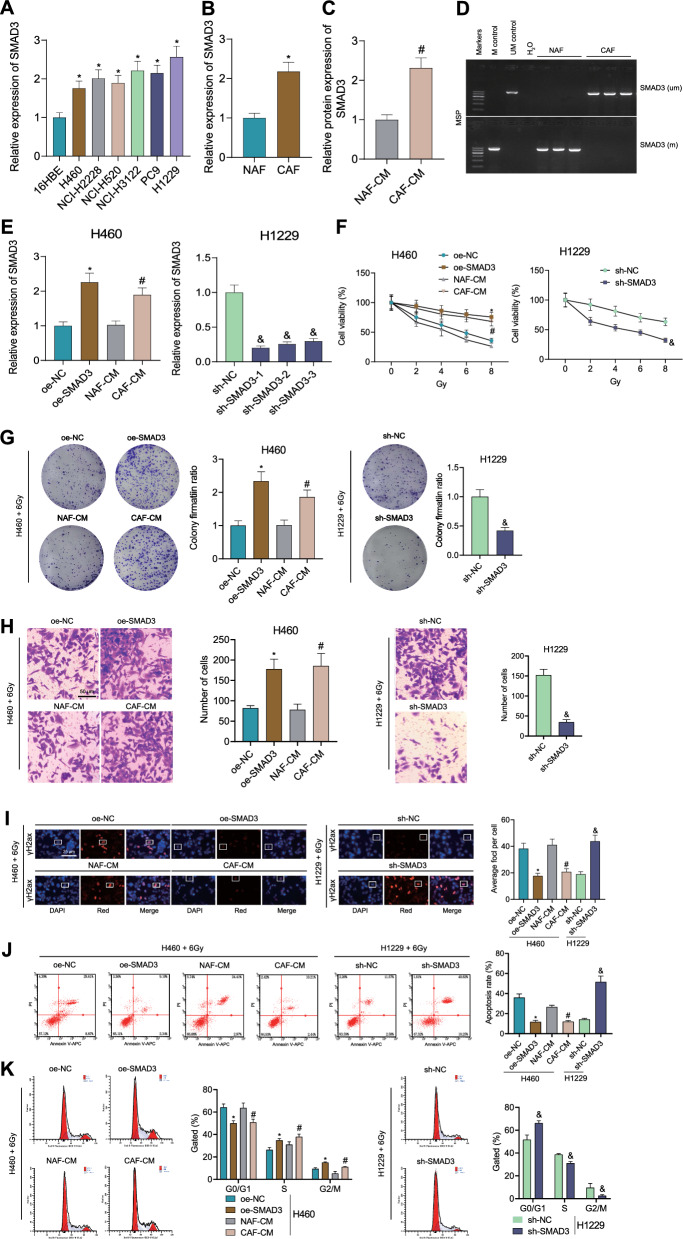
DNA hypomethylation of the SMAD3 promoter region in CAFs leads to increased radioresistance of NSCLC cells. **A** The expression of SMAD3 in NSCLC cell lines and human bronchial epithelial cells 16HBE measured by RT-qPCR. **B** The expression of SMAD3 in NAFs and CAFs measured by RT-qPCR. **C** Western blot of SMAD3 protein in the CAF-CM and NAF-CM. **D** The DNA methylation level of the SMAD3 promoter region in NAFs and CAFs measured by MSP-PCR. **E** The expression of SMAD3 in H460 cells treated with oe-SMAD3 or CAF-CM and in H1229 cells treated with sh-SMAD3 measured by RT-qPCR. **F** Viability of H460 and H1229 cells treated with oe-SMAD3 or sh-SMAD3, respectively, following exposure to different doses of X-ray radiation. **G** Proliferation of H460 and H1229 cells treated with oe-SMAD3 or sh-SMAD3, respectively, following exposure to 6 Gy X-ray radiation. **H** Invasion of H460 and H1229 cells treated with oe-SMAD3 or sh-SMAD3, respectively, following exposure to 6 Gy X-ray radiation. **I** γH2ax fluorescence in H460 cells treated with oe-SMAD3 or CAF-CM and in H1229 cells treated with sh-SMAD3 following exposure to 6 Gy X-ray radiation. **J** Apoptosis of H460 cells treated with oe-SMAD3 or CAF-CM and of H1229 cells treated with sh-SMAD3 following exposure to 6 Gy X-ray radiation determined by flow cytometry. **K** Cell cycle distribution of H460 cells treated with oe-SMAD3 or CAF-CM and of H1229 cells treated with sh-SMAD3 following exposure to 6 Gy X-ray radiation determined by flow cytometry. *p < 0.05 vs. 16HBE, NAF, or oe-NC groups. ^#^*p* < 0.05 vs. NAF-CM group. ^&^*p* < 0.05 vs. sh-NC group. Cell experiments were repeated three times

The above bioinformatics results were then validated in the in vitro experiments. RT-qPCR results showed higher SMAD3 expression in NSCLC cells compared with human bronchial epithelial cells 16HBE, with the highest expression noted in H1299 cells and the lowest expression in H460 cells (Fig. 5A). Thus, H1299 and H460 cells were used for subsequent experiments.

CCK-8 results demonstrated a decline in NSCLC cell viability following exposure to different doses of X-ray radiation compared with 16HBE cells (Additional file 3: Fig. S3A), which was consistent with SMAD3 expression in cells. This indicates that SMAD3 is highly expressed in NSCLC cells and correlated with radioresistance.

Human lung NAFs and CAFs were successfully isolated from fresh NSCLC and adjacent normal tissues (Additional file 3: Fig. S3B, C). RT-qPCR and Western blot results showed higher SMAD3 expression in CAFs and the CAF-CM versus the NAFs and NAF-CM (Fig. 5B, C). Additionally, MSP-PCR results revealed that the DNA methylation level of the SMAD3 promoter region was reduced in CAFs compared with the NAFs (Fig. 5D). This indicates that the SMAD3 promoter region is hypomethylated but SMAD3 expression was upregulated in CAFs.

Next, we transfected SMAD3 into NSCLC cells or added CAF-conditioned media (CM) to the NSCLC cells. The results of RT-qPCR (Fig. 5E) showed that SMAD3 expression was upregulated in NSCLC cells with oe-SMAD3 transfection and CAFs-CM treatment, while downregulated with sh-SMAD3 (subsequently using sh-SMAD3-1 for experiments). It should be noted that extracellular SMAD3 molecules do not directly enter NSCLC cells to exert their function. We speculate that SMAD3 binds to ITGA6 on the membrane of NSCLC cells. Subsequently, we collected cells for Western blotting experiments, and in the CAF-CM group, we observed an increase in SMAD3 protein levels in the cell lysates (Fig. 5E). This observation, combined with our previous finding of a significant increase in SMAD3 protein content in CAFs-CM (Fig. 5C), and the characteristic of ITGA6 as a transmembrane adhesive receptor protein, supports our hypothesis. Additionally, besides serving as a positive control, the overexpression of SMAD3 in NSCLC cells further confirms the specificity of the phenotypic results induced by CAFs-CM and excludes the influence of other cytokines present in CAFs-CM.

Using different intensities of radiation on NSCLC cells, the CCK8 results showed (Fig. 5F) that high expression of SMAD3 in NSCLC cells enhanced cell viability, while silencing SMAD3 resulted in decreased cell viability. Further treatment of cells with a dose of 6 Gy, colony formation, and Transwell experiments revealed (Fig. 5G–H) that high expression of SMAD3 enhanced colony formation and invasion ability, while silencing SMAD3 had the opposite effect. DNA double-strand breaks in NSCLC cells were assessed by immunofluorescence staining of γH2AX, a marker for DNA damage evaluation. The results of γH2AX immunofluorescence staining and flow cytometry (Fig. 5IK) showed that in the group with high SMAD3 expression, the intensity of γH2AX fluorescence decreased, indicating reduced DNA damage and decreased apoptosis. Additionally, the proportion of cells in the G0/G1 phase decreased, while cells in the S phase and cell division increased. The results were opposite when SMAD3 was silenced.

Cumulatively, DNA hypomethylation of the SMAD3 promoter region in CAFs can induce the radioresistance of NSCLC cells.

### SMAD3 from CAFs activates the ITGA6/PI3K/Akt pathway in NSCLC cells

**Fig. 6:**
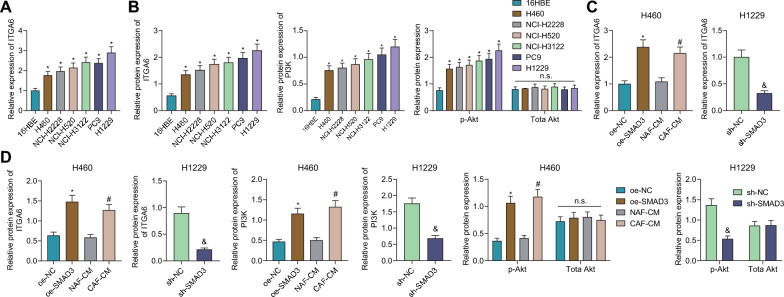
SMAD3 from CAFs promotes activation of the ITGA6/PI3K/Akt pathway in NSCLC cells**.** **A** ITGA6 mRNA expression in NSCLC cell lines and 16HBE cells measured by RT-qPCR. **B** Western blot of ITGA6 and PI3K/Akt pathway-related proteins in NSCLC cell lines and 16HBE cells. **C** ITGA6 mRNA expression in H460 cells treated with oe-SMAD3 or CAF-CM and in H1229 cells treated with sh-SMAD3 by RT-qPCR. **D** Western blot of ITGA6 and PI3K/Akt pathway-related proteins in H460 cells treated with oe-SMAD3 or CAF-CM and in H1229 cells treated with sh-SMAD3. *p < 0.05 vs. 16HBE or oe-NC group. n.s. indicates not significant. ^#^*p* < 0.05 vs. NAF-CM group. ^&^*p* < 0.05 vs. sh-NC group. Cell experiments were repeated three times

Validation of SMAD3 in CAFs activating the ITGA6/PI3K/Akt pathway in NSCLC cells was the next focus. RT-qPCR and Western blot results unveiled increased ITGA6 mRNA and protein expression in NSCLC cells compared with 16HBE cells, with the highest expression found in H1299 cells and the lowest in H460 cells. In addition, PI3K protein expression and Akt phosphorylation level were elevated while total Akt protein expression remained unchanged (Fig. 6A, B).

As depicted in Fig. 6C, D, treatment with oe-SMAD3 or CAF-CM enhanced mRNA and protein expression of ITGA6, PI3K protein expression and Akt phosphorylation level, without altering total Akt protein expression. In contrast, SMAD3 silencing caused opposite results except for the unchanged total Akt protein expression.

Together, SMAD3 from CAFs can activate the ITGA6/PI3K/Akt pathway in NSCLC cells.

### Activation of the ITGA6/PI3K/Akt pathway promotes radioresistance of NSCLC cells

**Fig. 7:**
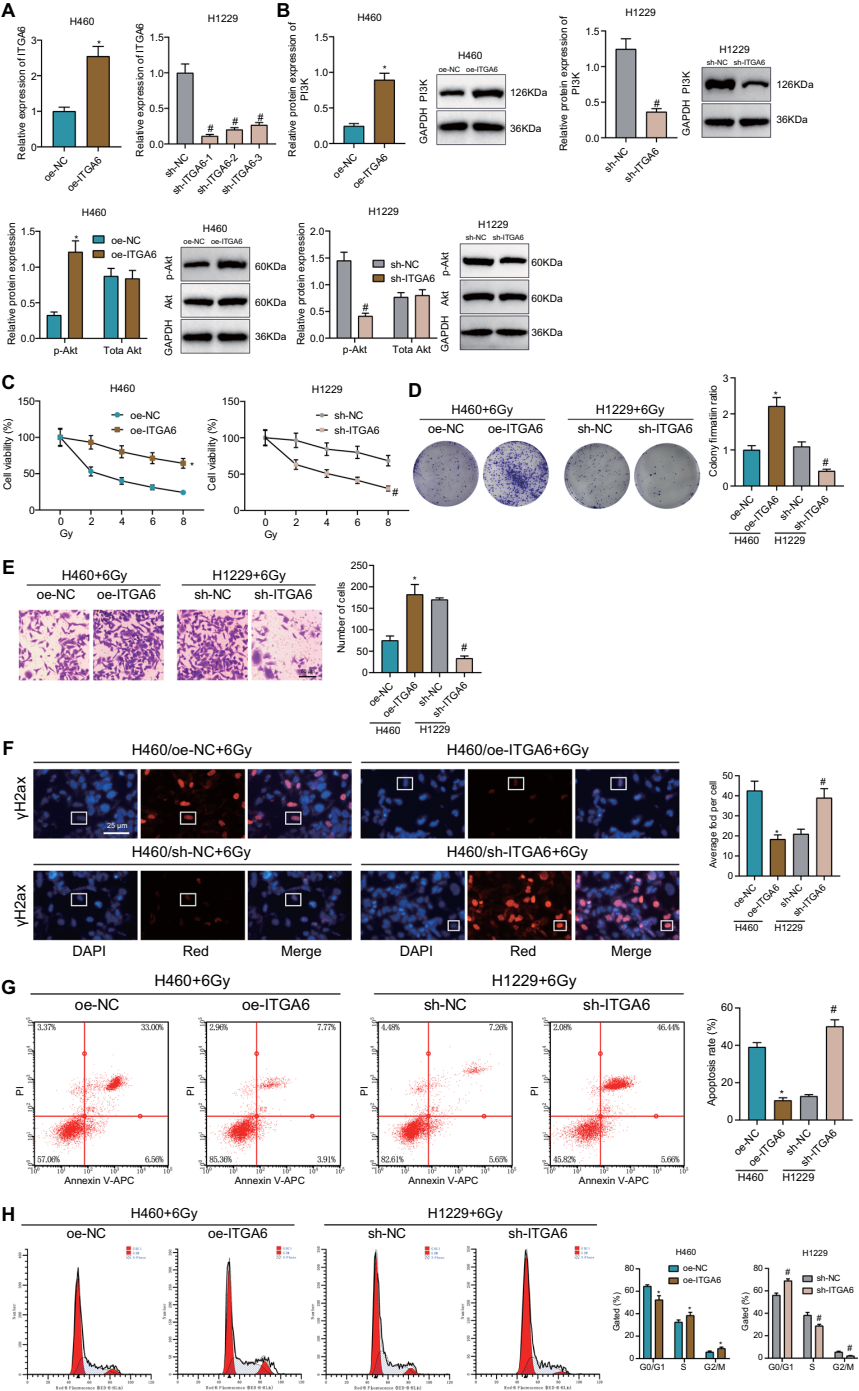
ITGA6/PI3K/Akt pathway exerts promoting function in the radioresistance of NSCLC cells**.** **A** ITGA6 mRNA expression in H460 and H1229 cells treated with oe-ITGA6 or sh-ITGA6, respectively, measured by RT-qPCR. **B** Western blot of PI3K/Akt pathway-related proteins in H460 and H1229 cells treated with oe-ITGA6 or sh-ITGA6, respectively. **C** Viability of H460 and H1229 cells treated with oe-ITGA6 or sh-ITGA6, respectively, following exposure to different doses of X-ray radiation. **D** Proliferation of H460 and H1229 cells treated with oe-ITGA6 or sh-ITGA6, respectively, following exposure to 6 Gy X-ray radiation. **E** Invasion of H460 and H1229 cells treated with oe-ITGA6 or sh-ITGA6, respectively, following exposure to 6 Gy X-ray radiation. **F** γH2ax fluorescence in H460 and H1229 cells treated with oe-ITGA6 or sh-ITGA6, respectively, following exposure to 6 Gy X-ray radiation. **G** Apoptosis of H460 and H1229 cells treated with oe-ITGA6 or sh-ITGA6, respectively, following exposure to 6 Gy X-ray radiation determined by flow cytometry. **H** Cell cycle distribution of H460 and H1229 cells treated with oe-ITGA6 or sh-ITGA6, respectively, following exposure to 6 Gy X-ray radiation determined by flow cytometry. *p < 0.05 vs. oe-NC group. ^#^*p* < 0.05 vs. sh-NC group. Cell experiments were repeated three times

Then, we intended to verify the effect of the ITGA6/PI3K/Akt pathway on radioresistance of NSCLC cells. RT-qPCR results indicated augmented ITGA6 expression in H460 cells treated with oe-ITGA6 while sh-ITGA6 in H1229 cells downregulated ITGA6 expression, with the sh-ITGA6-1 showing the most obvious downregulation (Fig. 7A) and thus used for the subsequent assay. In Fig. 7B, overexpression of ITGA6 enhanced PI3K protein expression and Akt phosphorylation level, without altering total Akt protein expression but silencing of ITGA6 downregulated PI3K protein expression and Akt phosphorylation level, in addition to the unaffected total Akt protein expression. These results support that ITGA6 positively regulates the PI3K/Akt pathway.

Furthermore, cell viability was promoted in the presence of ITGA6 overexpression following exposure to different doses of X-ray radiation, whereas an opposite result was caused by silencing of ITGA6 (Fig. 7C). Upon exposure to 6 Gy X-ray radiation, cell colony formation and invasion were enhanced in response to ITGA6 overexpression, which was negated by silencing of ITGA6 (Fig. 7D, E). Moreover, ITGA6 overexpression weakened γH2ax fluorescence intensity, and reduced DNA damage, cell apoptosis, and G0/G1 phase-arrested cells, while increasing S phase-arrested cells and cell division upon exposure to 6 Gy X-ray radiation; contrary results were evident following ITGA6 silencing (Fig. 7F–H).

These lines of evidence suggest that activation of the ITGA6/PI3K/Akt pathway may promote the radioresistance of NSCLC cells.

### SMAD3 from CAFs increases NSCLC cell radioresistance by activating the ITGA6/PI3K/Akt pathway

**Fig. 8:**
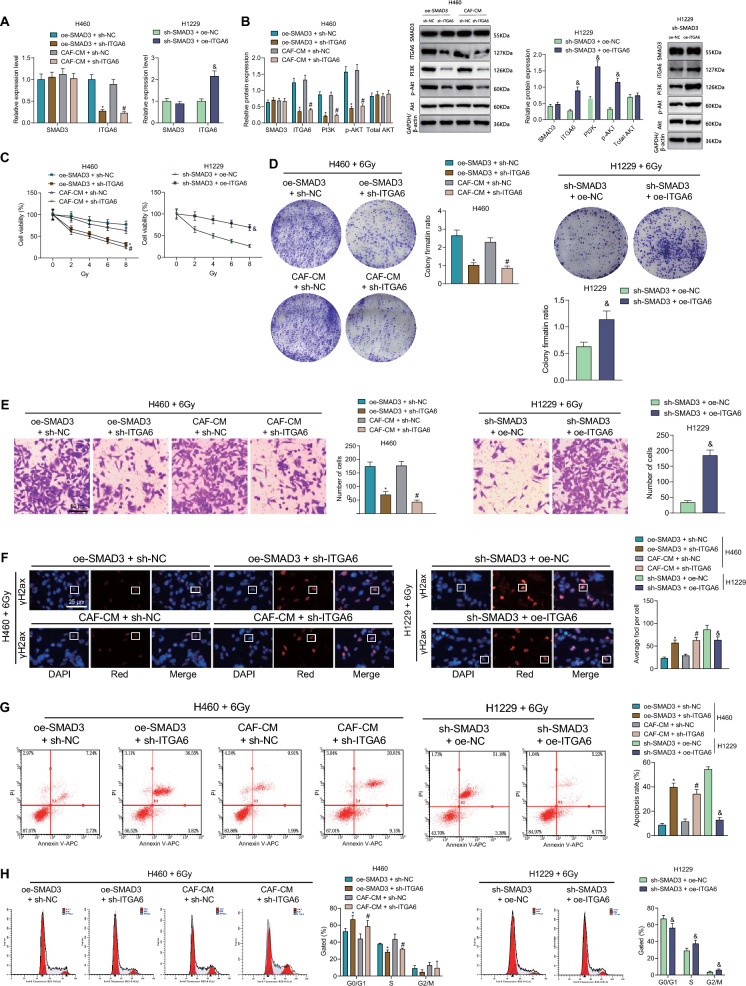
SMAD3 from CAFs reduces the radiosensitivity of NSCLC cells by activating the ITGA6/PI3K/Akt pathway. **A** The expression of SMAD3 and ITGA6 in H460 cells treated with sh-ITGA6 + oe-SMAD3 or sh-ITGA6 + CAF-CM and in the H1229 cells treated with sh-SMAD3 + oe-ITGA6 determined by RT-qPCR. **B** Western blot of SMAD3, ITGA6 and the PI3K/Akt pathway-related proteins in H460 cells treated with sh-ITGA6 + oe-SMAD3 or sh-ITGA6 + CAF-CM and in the H1229 cells treated with sh-SMAD3 + oe-ITGA6. **C** Viability of H460 cells treated with sh-ITGA6 + oe-SMAD3 or sh-ITGA6 + CAF-CM and of H1229 cells treated with sh-SMAD3 + oe-ITGA6 following exposure to 6 Gy X-ray radiation measured by CCK-8. **D** Colony formation of H460 cells treated with sh-ITGA6 + oe-SMAD3 or sh-ITGA6 + CAF-CM and of H1229 cells treated with sh-SMAD3 + oe-ITGA6, following exposure to 6 Gy X-ray radiation. **E** Invasion of H460 cells treated with sh-ITGA6 + oe-SMAD3 or sh-ITGA6 + CAF-CM and of H1229 cells treated with sh-SMAD3 + oe-ITGA6, following exposure to 6 Gy X-ray radiation. **F** γH2ax fluorescence in H460 cells treated with sh-ITGA6 + oe-SMAD3 or sh-ITGA6 + CAF-CM and in H1229 cells treated with sh-SMAD3 + oe-ITGA6, following exposure to 6 Gy X-ray radiation. **G** Apoptosis of H460 cells treated with sh-ITGA6 + oe-SMAD3 or sh-ITGA6 + CAF-CM and of H1229 cells treated with sh-SMAD3 + oe-ITGA6, following exposure to 6 Gy X-ray radiation determined by flow cytometry. **H** Cell cycle distribution of H460 cells treated with sh-ITGA6 + oe-SMAD3 or sh-ITGA6 + CAF-CM and of H1229 cells treated with sh-SMAD3 + oe-ITGA6, following exposure to 6 Gy X-ray radiation determined by flow cytometry. *p < 0.05 vs. oe-SMAD3 + sh-NC group. ^#^*p* < 0.05 vs. CAF-CM + sh-NC group. ^&^*p* < 0.05 vs. sh-SMAD3 + oe-NC group. Cell experiments were repeated three times

The results of RT-qPCR and Western blot showed unaltered SMAD3 expression and total Akt protein expression yet reduced ITGA6 expression, PI3K protein expression and Akt phosphorylation level in the H460 cells co-treated with sh-ITGA6 and oe-SMAD3 or sh-ITGA6 and CAF-CM than SMAD3 overexpression or CAF-CM alone. In the H1229 cells treated with sh-SMAD3 + oe-ITGA6, SMAD3 expression and total Akt protein expression showed no changes and ITGA6 expression, PI3K protein expression and Akt phosphorylation level were promoted (Fig. 8A, B).

Following exposure to different doses of X-ray radiation, cell viability was impaired upon treatment with sh-ITGA6 + oe-SMAD3 whereas an opoiste result was found upon treatment with sh-SMAD3 + oe-ITGA6 (Fig. 8C). In addition, upon exposure to 6 Gy X-ray radiation, cell colony formation and invasion as well as S phase-arrested cells and cell division were reduced but γH2ax fluorescence intensity, DNA damage, cell apoptosis, and G0/G1 phase-arrested cells were increased in response to treatment with sh-ITGA6 + oe-SMAD3, the effect of which was reversed by sh-SMAD3 + oe-ITGA6 (Fig. 8D–H).

Taken together, SMAD3 from CAFs can enhance the radioresistance of NSCLC cells by activating the ITGA6/PI3K/Akt pathway.

### SMAD3 from CAFs promotes tumor growth and radioresistance of NSCLC cells by activating the ITGA6/PI3K/Akt pathway in vivo

**Fig. 9:**
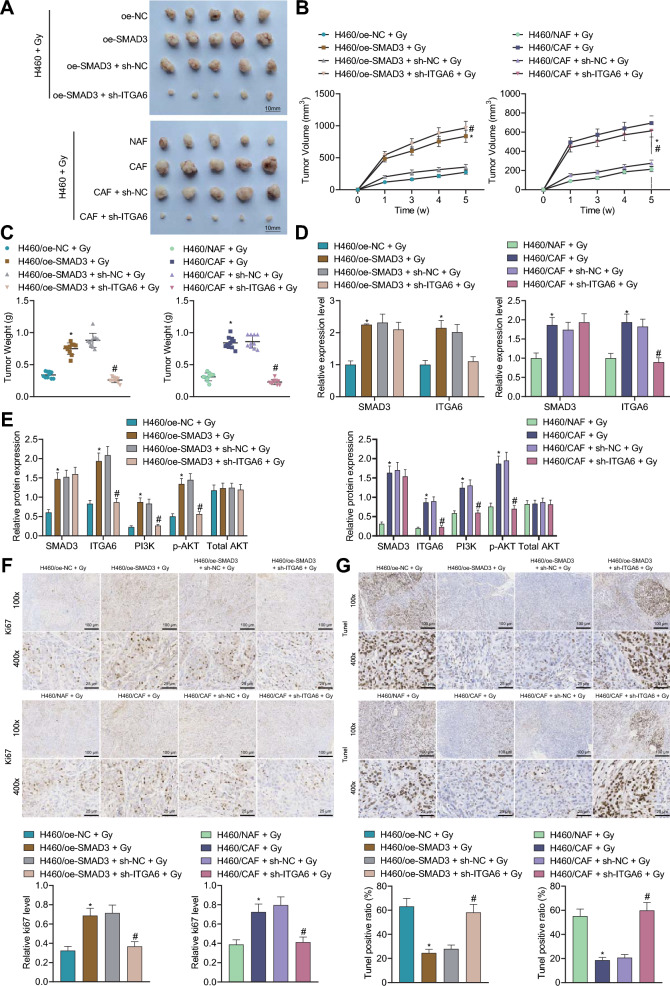
SMAD3 from CAFs induces tumor growth by activating the ITGA6/PI3K/Akt pathway in vivo. Nude mice were treated with H460/oe-NC + Gy, H460/NAF + Gy, H460/oe-SMAD3 + Gy, H460/CAFs + Gy, H460/oe-SMAD3 + sh-NC + Gy, H460/CAFs + sh-NC + Gy, H460/oe-SMAD3 + sh-ITGA6 + Gy and H460/CAFs + sh-ITGA6 + Gy. **A** Schematic diagram of xenografts in nude mice. **B** Tumor volume of nude mice. **C** Tumor weight of nude mice. **D** Expression of SMAD3 and ITGA6 in tumor tissues of nude mice determined by RT-qPCR. **E** Western blot of SMAD3, ITGA6 and the PI3K/Akt pathway-related proteins in tumor tissues of nude mice. **F** Cell proliferation in tumor tissues of mice determined by immunohistochemical staining for Ki67. **G** Cell apoptosis in tumor tissues of mice determined by TUNEL staining. n = 10. *p < 0.05 vs. H460/oe-NC + Gy or H460/NAF + Gy group. ^#^*p* < 0.05 vs. H460/oe-SMAD3 + sh-NC + Gy or H460/NAF + sh-NC + Gy group

Finally, we proceeded to characterize whether SMAD3 from CAFs promoted the radioresistance of NSCLC cells through activation of ITGA6/PI3K/Akt pathway in vivo. As displayed in Fig. 9A–C, tumor volume and weight were increased in mice treated with H460/oe-SMAD3 + Gy or H460/CAFs + Gy, and conversely, H460/oe-SMAD3 + sh-ITGA6 + Gy or H460/CAFs + sh-ITGA6 + Gy led to reduced tumor volume and weight.

The results of RT-qPCR and Western blot demonstrated an enhancement in the mRNA and protein expression of SMAD3, ITGA6, PI3K protein expression and Akt phosphorylation level yet unchanged total Akt protein expression in the tumor tissues of mice treated with H460/oe-SMAD3 + Gy or H460/CAFs + Gy. In contrast, H460/oe-SMAD3 + sh-ITGA6 + Gy or H460/CAFs + sh-ITGA6 + Gy resulted in opposite results in addition to unaltered total Akt protein expression (Fig. 9D, E).

Furthermore, data shown in Fig. 9F, G explained high percentage of Ki67-positive tumor cells and low cell apoptosis in response to H460/oe-SMAD3 + Gy or H460/CAFs + Gy. However, these effects were reversed following treatment with H460/oe-SMAD3 + sh-ITGA6 + Gy or H460/CAFs + sh-ITGA6 + Gy.

Overall, the above results indicate that SMAD3 from CAFs might favor NSCLC tumor growth in vivo and enhances tumor radioresistance via activation of the ITGA6/PI3K/Akt pathway.

## Discussion

Investigating the molecular pathways and mechanisms that facilitate interaction between CAFs and tumor cells during radiotherapy is crucial for enhancing clinical outcomes in patients with NSCLC [36]. This knowledge is foundational for developing targeted therapies to augment the efficacy of radiotherapy in NSCLC, ultimately aiming to improve patient prognosis [37]. The current study elucidates the contributory role of SMAD3 in NSCLC radioresistance, delineating the mechanism wherein enforced SMAD3 expression activates the ITGA6/PI3K/Akt pathway, thereby intensifying NSCLC radioresistance. These findings underscore the potential of SMAD3 as a targeted therapy and a prognostic indicator for NSCLC patients.

The present findings position SMAD3 as an independent prognostic factor in NSCLC, with its heightened expression correlating with poor patient prognosis. Concurrently, recent research has indicated that phosphorylation of SMAD3 in immune cells is predictive of survival in early-stage NSCLC patients, associating increased phosphorylation with reduced survival [38]. Moreover, elevated SMAD3 expression has been identified as an independent marker for better overall survival in NSCLC patients [19]. This study also reveals high SMAD3 expression by CAFs within the tumor microenvironment, correlating with promoter hypermethylation [19]. In breast cancer, CAFs predominantly activate the SMAD pathway through paracrine TGF-b1 [39]. Elevated SMAD3 mRNA expression has been detected in adenocarcinoma-associated fibroblasts [40]. Notably, aberrant genomic DNA methylation in NSCLC tumor-associated fibroblasts is a critical regulator of tumor progression, with SMAD3 promoter hypermethylation associated with reduced gene expression [41]. Evidence suggests that inhibiting SMAD3 in CAFs diminishes its activation and secretion of extracellular matrix components, thereby curtailing tumor cell survival and enhancing radiosensitivity [42]. Thus, strategies to inhibit SMAD3 activity in CAFs may present a novel approach to augment radiotherapy efficacy in NSCLC and improve patient clinical prognosis [19].

This study also indicates that CAF-derived SMAD3 heightens the radioresistance of NSCLC cells, associated with the activation of the ITGA6/PI3K/Akt pathway. CAFs secrete various immunomodulatory soluble factors, including cytokines, growth factors, and chemokines, which directly impact tumor immunity and inflammation, playing a pivotal role in tumor radioresistance [43]. Aligning with these findings, SMAD3 expression significantly differs between radiation-resistant and radiation-sensitive patients; SMAD3 knockdown markedly inhibits cell growth and boosts radiosensitivity of lung adenocarcinoma cells both in vitro and in vivo [44]. Moreover, ITGA6 knockdown is responsible for the inhibition of the TGF-β1/SMAD pathway in choriocarcinoma cells [45]. ITGA6 has emerged as a significant predictive marker of radioresistance, with its silencing attenuating radioresistance in head and neck cancer cells [46]. Data also suggest that ITGA6 inhibition counters radiotherapy resistance in mesenchymal glioblastoma stem-like cells [47]. Forced ITGA6 expression elevates PI3K and Akt phosphorylation levels in oral squamous cell carcinoma cells, but its inhibition impairs the activity of the PI3K/Akt pathway [48]. Additionally, ITGA6 overexpression promotes radioresistance in breast cancer cells by activating the PI3K/Akt pathway [49]. Previous results also indicate that PI3K/Akt pathway inhibition enhances the sensitivity of NSCLC cells to ionizing radiation, suggesting that PI3K/Akt inhibitors may be a strategy to reduce NSCLC radioresistance [50]. Notably, SMAD3 overexpression leads to significant activation of the PI3K/Akt pathway in vascular smooth muscle cells and a rat carotid artery injury model [51].

**Fig. 10:**
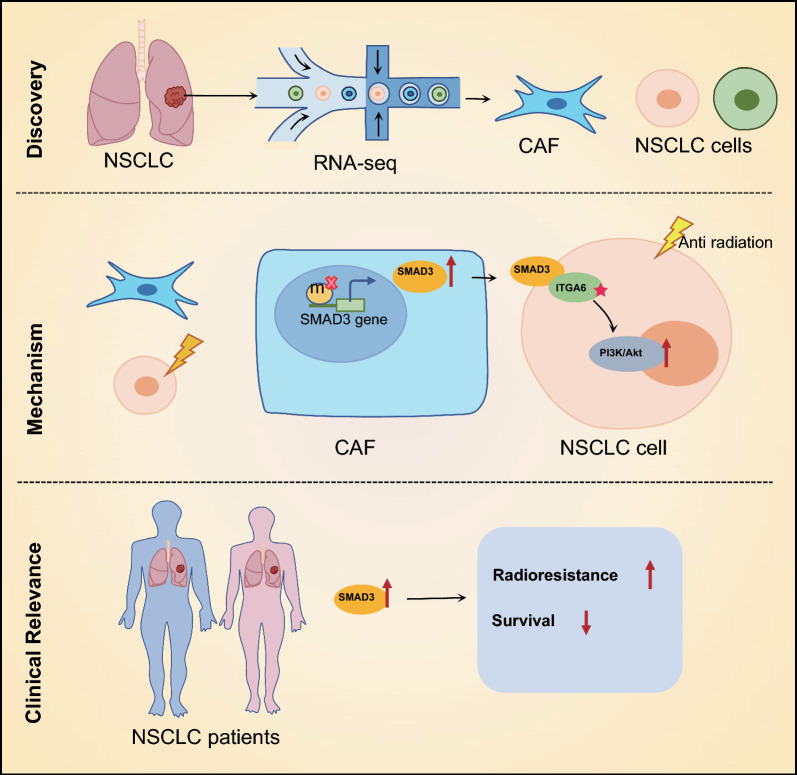
The mechanism graph of the regulatory network and function of SMAD3 in radioresistance of NSCLC. SMAD3 is significantly enriched in CAFs, which may promote the radioresistance of NSCLC cells by activating the ITGA6/PI3K/Akt pathway

In summary, our work has provided the first evidence of the radioprotective effect of SMAD3 on NSCLC cells. Furthermore, our study has demonstrated a novel regulatory pathway of SMAD3 in NSCLC cells, namely the SMAD3/ITGA6/PI3K/Akt signaling pathway, as illustrated in Fig. 10. In stromal cells, a decrease in the methylation level of the SMAD3 gene promoter region leads to an overexpression of SMAD3 molecules. These SMAD3 molecules are secreted into the tumor microenvironment and interact with ITGA6 on the membrane of NSCLC cells, resulting in its hyperactivation. Subsequently, ITGA6 further activates the downstream PI3K/Akt signaling pathway, leading to increased cell proliferation, enhanced cell survival, and increased invasion capability, thus enhancing the radiation resistance of NSCLC.

Our research suggests that developing drugs targeting the SMAD3 and its downstream ITGA6/PI3K/Akt axis may be an effective strategy for NSCLC prognosis and treatment. SMAD3 has been identified as a potential biomarker for NSCLC prognosis and a driving factor for radiation resistance. These findings could lead to novel targeted treatment strategies aimed at inhibiting the SMAD3 and ITGA6/PI3K/Akt pathways to improve radiotherapy efficacy and patient prognosis. Additionally, the development of targeted therapies to alleviate radiation resistance mediated by CAFs may reduce treatment-related toxicity and improve the quality of life for patients. By increasing the sensitivity of NSCLC cells to radiotherapy, it is possible to reduce the required radiation doses and consequently lower the incidence of treatment-related adverse events in NSCLC patients. Ultimately, by elucidating the molecular mechanisms underlying the role of CAFs in the development of radiation resistance, new treatment strategies can be developed to improve clinical outcomes and reduce the incidence of treatment-related complications in NSCLC patients. Furthermore, the molecular basis of the relationship between the overexpression of SMAD3 and the low DNA methylation in its promoter region needs further investigation. Therefore, future studies can focus on expanding the sample size and conducting more detailed molecular analyses to gain a better understanding of the role of SMAD3 in NSCLC radioresistance.

Additionally, there are certain limitations to this study. Firstly, the precise targeting and regulation of molecules such as SMAD3 and ITGA6 in patients with lung cancer remains relatively challenging given the current technological level. Furthermore, the tumor microenvironment is highly complex, and whether the designed drugs can achieve the expected regulatory effects within patients and the corresponding optimal concentrations still require further scientific exploration. Moreover, if systemic treatment methods are employed, the potential side effects of the relevant drugs on a systemic level were not addressed in this study and need to be further investigated. It is also worth noting that the theoretical basis provided by the conclusions of this study is only one possible strategy for reducing the radioresistance of NSCLC cells, and it still remains an auxiliary means for lung cancer prevention or treatment. Therefore, for lung cancer disease, more scientific research is still needed to clarify its pathological mechanisms and provide a more direct theoretical foundation for novel preventive or therapeutic methods. Lastly, it is necessary to further explore whether the SMAD3/ITGA6 signaling pathway has similar biological functions in other types of cancer.

### Supplementary Information

**Additional file 1: Figure S1.** Differential analysis of datasets from GEO database. **A** A volcano map of DEGs between control samples and NSCLC samples in five datasets from GEO database (GSE2514, GSE18842, GSE21933, GSE31552 and GSE44077). Green indicates the downregulated genes, red indicates the upregulated genes, and black indicates genes with no differential expression. **B** Venn diagram of the upregulated DEGs in the five datasets. **C** Venn diagram shows the downregulated DEGs in the five datasets. **D** A volcano plot of the DEGs between the radioresistant and radiosensitive NSCLC cell lines in the GSE20549 dataset. Green indicates the downregulated genes, red indicates the upregulated genes, and black indicates genes with no differential expression.

**Additional file 2: Figure S2.** Correlation of SMAD3 expression and methylation at promoter CpG sites. **A** The DNA methylation level of 29 SMAD3 promoter CpG sites in TCGA-LUAD. **B** The DNA methylation level of 29 SMAD3 promoter CpG sites in TCGA-LUSC. **C** 18 CpG sites adversely correlated with SMAD3 expression in TCGA-LUAD. **D** 17 CpG sites negatively correlated with SMAD3 expression in TCGA-LUSC.

**Additional file 3: Figure S3.** Radioresistance of NSCLC cells and identification of NAFs and CAFs. **A** CCK-8 detection of viability of 16HBE and NSCLC cells following exposure to different doses of X-ray radiation. **B** Morphology of primary fibroblasts under a microscope. **C** Immunofluorescence staining of α-SMA and FAP proteins in NAFs and CAFs. Cell experiments were repeated three times. *p < 0.05 vs. 16HBE cells.

**Additional file 4: Table S1. **Clinical characteristics of 120 NSCLC patients. **Table S2.** Primer sequences of qRT-PCR. **Table S3.** The primer sequences for MSP. **Table S4.** shRNA sequences.

## Data Availability

The datasets generated and/or analysed during the current study are available in the manuscript and supplementary materials.
