# Supplementary material for: Single-cell transcriptomic sequencing data reveal aberrant DNA methylation in SMAD3 promoter region in tumor-associated fibroblasts affecting molecular mechanism of radiosensitivity in non-small cell lung cancer
Source: J Transl Med. 2024 Mar 16;22:288. doi: 10.1186/s12967-024-05057-2 (PMC10944599; doi:10.1186/s12967-024-05057-2)
Supplement: Supplementary file 4 — Additional file 4: Table S1. Clinical characteristics of 120 NSCLC patients. Table S2. Primer sequences of qRT-PCR. Table S3. The primer sequences for MSP. Table S4. shRNA sequences. [file 12967_2024_5057_MOESM4_ESM.docx]

**Table S1** **Clinical characteristics of 120 NSCLC patients**

| Characteristics | All patients (n = 120) |
| --- | --- |
| Age (years) |  |
| Median (range) | 62 (29-84) |
| Gender (%) |  |
| Male | 54 |
| Female | 66 |
| Histology (%) |  |
| Adenocarcinoma | 104 (86.67%) |
| Squamous cell carcinoma | 14 (11.67%) |
| Atypical carcinoid | 1 (0.83%) |
| Adenosquamous carcinoma | 1 (0.83%) |
| Large cell neuroendocrine carcinoma | 0 (0.00%) |
| pTMN stage (%) |  |
| I | 88 (73.33%) |
| II | 7 (5.83%) |
| III | 15 (12.50%) |
| IV | 10 (8.33%) |
| T stage (%) |  |
| T1–T3 | 116 (96.67%) |
| T4 | 4 (3.33%) |
| N stage (%) |  |
| N0–N1 | 98 (81.67%) |
| N2-N3 | 21 (17.50%) |
| Nx | 1 (0.83%) |

**Table S2** Primer sequences of qRT-PCR

| Gene | Sequence |
| --- | --- |
| SMAD3 | Forward: 5'-TGAAGCGCACTGACCATAAGA-3' |
|  | Reverse: 5'-GGACTCAAACGTGGTTGCTC-3' |
| ITGA6 | Forward: 5'-ACACAGCATTGTATATGTGAAGCA-3' |
|  | Reverse: 5'-CCGAATCCCATTGCTTTGGC-3' |
| GAPDH | Forward: 5'-AATGGGCAGCCGTTAGGAAA-3' |
|  | Reverse: 5'-GCGCCCAATACGACCAAATC-3' |

**Table S3** The primer sequences for MSP

| Gene | Sequence |
| --- | --- |
| SMAD3 (methylation) | Forward: 5'-TTTATAGGGTTTCGTAAAGCGTATC-3' |
|  | Reverse: 5'-GAATTCAAAACTAATTACCCACGAT-3' |
| SMAD3 (unmethylation) | Forward: 5'-TATAGGGTTTTGTAAAGTGTATTGG-3' |
|  | Reverse: 5'-AAATTCAAAACTAATTACCCACAAT-3' |

**Table S4** shRNA sequences

| shRNA | Sequence |
| --- | --- |
| sh-SMAD3-1 | 5'-GCCTCAGTGACAGCGCTATTT-3' |
| sh-SMAD3-2 | 5'-GGATTGAGCTGCACCTGAATG-3' |
| sh-SMAD3-3 | 5'-GAGCCTGGTCAAGAAACTCAA-3' |
| sh-ITGA6-1 | 5'-CCTCTCAGATTCAGTAACTAT-3' |
| sh-ITGA6-2 | 5'-CGAGAAGGAAATCAAGACAAA-3' |
| sh-ITGA6-3 | 5'-CGGATCGAGTTTGATAACGAT-3' |
